# Spatial and Temporal Patterns of Deforestation in Rio Cajarí Extrative Reserve, Amapá, Brazil

**DOI:** 10.1371/journal.pone.0051893

**Published:** 2012-12-17

**Authors:** Claudia Funi, Adriana Paese

**Affiliations:** 1 Programa de Pós Graduação em Biodiversidade Tropical, Universidade Federal do Amapá, Macapá, Brazil; 2 Instituto Estadual de Pesquisas do Amapá, Macapá, Brazil; 3 Conservação Internacional, Belo Horizonte, Brazil; University of Western Australia, Australia

## Abstract

The Rio Cajarí Extractive Reserve (RCER) is a sustainable use protected area located in Southern Amapá state, Brazil. This protected area is home to traditional agro-extractive families, but has been increasingly invaded by commercial agriculture producers. In this work, we test the hypothesis that the RCER implementation has distinctly affected spatial patterns of deforestation and rates of bare soil and secondary forest formation by the social groups occupying the protected area and its surrounding area. Detailed maps of vegetation cover and deforestation were elaborated, based on Landsat TM images from 1991, 1998, 2007 and 2008 and Linear Spectral Mixture Models. Based on an extensive fieldwork, patches were classified according to the agents causing deforestation and characterized with ten explanatory variables. A discriminant function analysis was used to identify homogeneous groups based on the data. Results show increased rates and distinct spatial patterns of deforestation by three groups: extractivists, non traditional commercial agriculture producers, and a less representative group constituted of miners, cattle and timber producers. In all analyzed dates, clearings by the extrativist community presented the highest total area and smaller average sizes and were located in close proximity to villages. Deforestation patches by the non-traditional group were exclusively associated with ombrophilous forests; these presented higher average sizes and proximity indexes, and showed increased aggregation and large cluster formation. No significant differences were observed in deforestation patterns by the three groups inside or outside the reserve.

## Introduction

As new targets for reducing deforestation in tropical forests are being set and as policy and incentives for reducing deforestation and forest degradation in developing countries are being discussed [1], [2], [3], a clearer understanding of the drivers of deforestation inside and outside protected areas is still needed. Moreover, there is still a need to quantify the effects of improved forest management on carbon retention [4].

Predictive models of the effects of infrastructure development and conservation policies in the Brazilian Amazon have demonstrated the contribution of the existing protected areas to regional climate regulation, [5] and their potential role in reducing CO_2_ emissions from deforestation [6] and post clearing agricultural practices [7]. Although coarse-scale global and regional assessments have demonstrated the effectiveness of protected areas in reducing the clearing of tropical forests [8], [9], [10], refinements to these analyses demonstrate a broader range of efficacy [11]. These refinements have incorporated biophysical and socioeconomic factors that affect the location of protected areas [12] or were conducted at temporal scales that match the patterns of human-caused disturbances [11].

Empirical evidence also shows that the projected scenarios may not be as optimistic as they may appear. Deforestation is known to be pervasive, and such pervasive deforestation is a result of diverging interests of different social and political groups. Small scale decisions by these groups affect land use inside and outside protected areas, hindering the implementation of the areas affected [13], [14].

Braziĺs Amazonian protected area network is constituted of municipal, state and federal sustainable use and strictly protected areas. These areas are unified under the National System of Protected Areas [15]. Protected areas in the Brazilian Amazon increased by 709,000 km^2^ between 2002 and 2009 [6]. Over the past 24 years, the average rate of forest clearing in the Brazilian Amazon was 16,341.71 km^2^ yr^−1^ [16]. In 2007, sustainable use areas and strictly protected areas represented 61% and 39%, respectively, of the total protected area in the region [17]. Sustainable use areas (e.g. extractive reserves, national forests, environmental protection areas) allow local people to develop subsistence agricultural activities, and partially restrict deforestation. In strict protection areas (e.g. parks, biological reserves, ecological stations), the exploitation of natural resources by human populations is prohibited [15]. Between 2000 and 2006, the average deforestation rate within protected areas was 1,520 km^2^ yr^−1^. The overall protected area network has helped maintain intact 98.6% of the forest cover inside the protected areas [11]. However, analyses of different categories or individual protected areas have indicated a broad range of efficacy. If clearings occur along their perimeters, extractive reserves are more vulnerable to forest cutting than other categories, including strictly protected areas and indigenous lands [9], [11].

Assessments of deforestation rates in the Brazilian Amazon are based primarily on the data from the PRODES Project (*Projeto de Monitoramento do Desmatamento na Amazônia Legal*) [6], [16], [9], [11]. The monitoring activities conducted by this project serve to detect deforestation patches above the 6.25 ha threshold. Although data from PRODES have been proved to be useful for detecting deforestation on agricultural frontiers, refinements to this methodology are necessary to detect land use change in more conservative scenarios. These more conservative settings include those involving low human population densities, those associated with vegetation other than upland forests (i.e., upland savannas and inundated savannas), or those associated with forest degradation (e.g. surface fire disturbance and selective logging) [18]. Evidence also demonstrates that protected areas in geographical proximity experiencing similar development pressure may show very different rates of forest clearing [11]. This information underscores the need for a clearer understanding of the social drivers of deforestation and occupation history in protected areas and their surrounding areas.

In this study, we performed a fine -grained analysis of the drivers of spatial and temporal patterns of deforestation by social groups in the Rio Cajarí Extractive Reserve (RCER) and its surrounding area. The RCER is a sustainable use protected area located in southern Amapá State, Brazil. Amapá is the Brazilian state with the highest proportion of native vegetation cover and the lowest average deforestation rate between 1998 and 2011 (58.77 ha) [19]. Approximately 70% of the area of this state is currently protected through designation as strictly protected and sustainable use areas, or as indigenous lands. The RCER is the sustainable use protected area (IUCN category VI protected area) with the greatest average deforestation rate in Amapá State [19]. The RCER was created, as were other extractive reserves in Brazil, with the primary objective of resolving land tenure issues and providing greater tenure security for local extractivist communities [20]. The RCER is home to traditional agroextractivist families, primarily Brazil-nut collectors, but it has been increasingly invaded by commercial agriculture producers.

This study analyzes a satellite image classification that allows the detection and mapping of finer scale spatial and temporal patterns of deforestation in a broad range of native vegetation covers. The pressures and drivers of deforestation are also examined in detail inside the RCER and in its immediate surroundings. We test the hypotheses that the RCER implementation has distinctly affected the spatial patterns of deforestation and rates of bare soil and secondary forest formation by the social groups occupying the protected area and its surrounding area.

## Materials and Methods

### Study Area

In order to account for potential threats to the reserve, our study area was defined as the Rio Cajarí Extractive Reserve area (RCER) and its immediate surroundings, including a buffer zone extending 5 km from the boundaries of the protected area. This definition of the buffer zone was selected to include all deforestation by extractivists settled in the RCER and its surrounding area.

The RCER was created through Federal Decree number 99,145, on March 12^th^, 1990 [20], and modified on September 30^th^, 1997 [21]. As an IUCN category VI protected area, the main goal of the RCER is to reconcile economic development with long-term environmental conservation, while improving the well-being of traditional populations.

**Figure 1:**
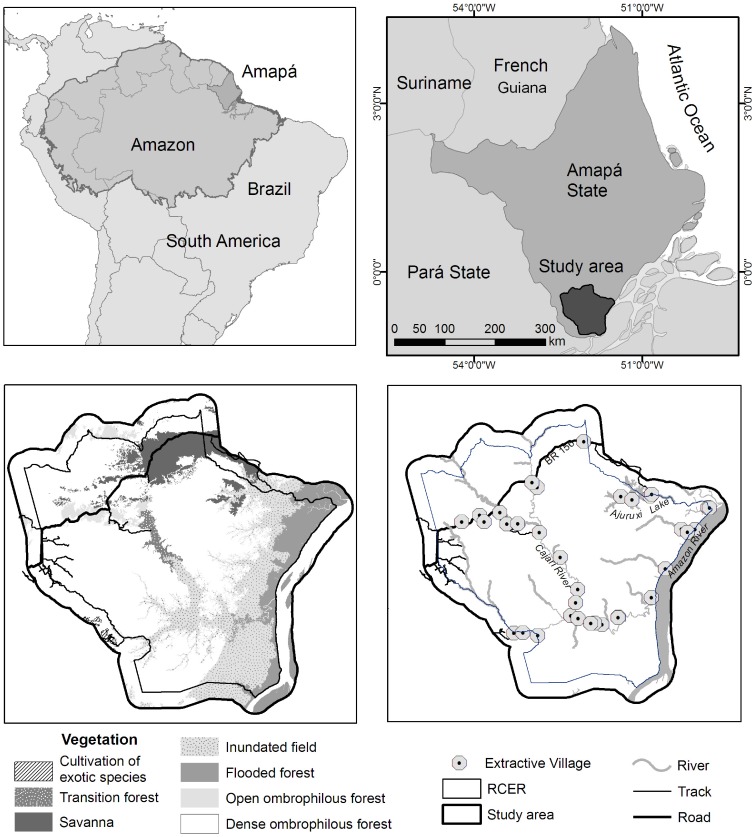
Location of the Amazon river basin and Amapá State in South America and Brazil (a); Location of the studied area in Amapá (b); Main vegetation cover in the Rio Cajarí extractive reserve and its immediate surrounding area(c); Location of extractivists’ villages terrestrial and fluvial accesses in the study area (d).

The total extent of our study area is 679,421.8 ha, of which 503,448.6 ha (74%) constitute the RCER area and 175,973.2 ha (26%) the reservés immediate surroundings. The study area is located from 0° 15′ S to 52° 25′ W and from 1° S to 51° 31′ W in the municipalities of Laranjal do Jari, Vitória do Jari and Mazagão, in Amapá State, Brazil. The RCER and its buffer zone are covered by dryland and flooded forests, upland savannas and inundated savannas [22]. The climate is classified as tropical wet, with an annual average temperature above 25°C and an average rainfall of 2,300 mm concentrated between December and June [23]. The elevation of the area ranges from 1 to 357 m [21]. The Amazon River forms the eastern boundary of the RCER. The northwestern portion of the reserve is crossed by an unpaved federal highway (BR 156). In absolute terms, the reserve shows the highest deforestation rate among all protected areas in Amapá State [19] (Figure 1).

In 1967, a large-scale enterprise, the Jari Project, was established in southern Amapá State. This project introduced large scale cultivation of exotic species (primarily *Eucalyptus sp*) for pulp production; cattle and buffalo husbandry, and included timber and mineral extraction [24]. As a result of the Jari Project implementation, large tracts of land were deforested, including areas long occupied by extractivist communities [25]. Intensified conflicts between the local communities and the contractors of the Jari Project resulted in the creation of extractivist settlements for the local communities through the agrarian reform agency (*Instituto Nacional de Colonização e Reforma Agraria* - INCRA) in 1987 (Decree No. 627 of 7/30/87), and in the creation of the Rio Cajari Extractive Reserve in 1990 [26]. Today, approximately 3,050 residents, distributed in 552 households, are located in the RCER in close proximity to roads and with access to water corridors [27]. In the RCER, the land is owned by the Federal Government. The extractivist communities hold joint usufruct rights over the extractive reserve. These rights may be transferred by inheritance. The RCER is entailed to the Chico Mendes Institute for Biodiversity Conservation (ICMBio), the governmental agency responsible for all federal protected areas in the country. The governance is decentralized and is conducted by the reservés Steering Committee (*Conselho Deliberativo*), composed of 23 representatives of the local, state and federal governments and local community associations.

A utilization plan, specifying the ways in which the community manages its resources was defined in 1997 as part of the land concession [15]. The utilization plan is a temporary instrument based on the residents` knowledge and experience. This plan will remain valid until a management plan is developed [28]. The RCER utilization plan includes the prohibition of commercial hunting and the recognition of common areas, such as rivers, lakes, pathways, banks and jointly managed areas in the reserve. The extraction of timber for commercial purposes is prohibited. According to the RCER utilization plan, each family is allowed to deforest areas no greater than 15 ha for the cultivation of subsistence crops (primarily manioc).

The main economic activity of the extractivist families living in the reserve is the exploration of non-timber forest products, primarily Brazil nuts (*Bertholletia excelsa*), the fruit and palm of açaí trees (*Euterpe oleracea*), and andiroba (*Carapa guianensis*). The rights of families to harvest non-timber forest products are dictated by the spatial pattern of the resource and the rules of the community. Families are allowed to explore areas known as ´*castanhais* ` or ´*colocações* `, which contain high densities of Brazil nut trees and are informally regarded as the familýs property. The rights to the exploration of *castanhais* are transferred to family members through inheritance or family division (marriage of a son). This process tends to reduce the sizes of exploration sites and to decrease familieś opportunities to thrive based solely on non-timber forest production.

### Deforestation Mapping

A high-resolution map of the vegetation cover was constructed based on Landsat TM5 satellite images from 1991 and linear spectral mixture models (LSMM) [29], [30]. Vegetation patches and classes were validated with the existing coarse-scale vegetation maps (IBGE) and 68 days of on-the-ground and aerial observations. Detailed maps of deforestation were generated for four dates with Landsat Thematic Mapper five images from 1991, 1998, 2007 and 2008. Landsat TM images were chosen for this analysis because of the temporal extent of cloud-free images available in the National Institute for Space Research (INPE) catalog [31]. We wanted to depict land use immediately after the reserve creation (1991), and at intermediate (1998) and recent dates (2007–2008). Amapá is located in the Intertropical Convergence Zone, where increased precipitation and cloud cover are common throughout the year. A mosaic of images from 2007–2008 was created to depict the recent pattern of land use because completely cloud-free scenes were not available for 2008. The deforestation maps were generated using linear spectral mixture models (LSMM) [29], [30] and on-screen digitizing. Vegetation, soil and shadow fraction images resulting from the LSMM allowed the identification of deforestation patches in distinct vegetation types.

### Agents Causing Deforestation

Deforestation polygons were classified as (a) agroextractive/traditional (AE), (b) non-traditional (NT), and (c) other occupants (OT) according to the agent causing deforestation. The AE deforestation polygons resulted from traditional community activities.

AE patches are deforestation patches opened by agroextractive families. Most of these families have been in the reserve area since the ´rubber boom` in the late 19^th^ century [32]- and remain involved with the extraction of timber and non-timber forest products and subsistence agricultural production. They possess substantial knowledge of the local natural resources and biological cycles. They practice non-mechanized agriculture and may use fire in these activities. Family members may serve as the labor force for family agricultural activities. The reserve utilization plan prohibits the use of contract employees for natural resources exploration or agricultural activities.

NT deforestation patches result from non-traditional land use practices. Non-traditional producers do not extract non timber forest products to complement their income. They practice intensive commercial agriculture and may have employees. The deforestation patches classified as Others (OT) include a less representative, but more heterogeneous group. This group consists of small cultivation sites, abandoned mining sites, or small patches opened by cattle and timber producers. These clearings are located on islands in the Amazon river in the area surrounding the RCER. The OT patches include clearings associated with mining activities in the northern part of the reserve, deforestation associated with the village of Jarilândia (located south of the reserve in the municipality of Vitória do Jari), or clearings associated with higher income farmers. Higher-income farmers are located exclusively in the proximity to the BR-156 road, outside the RCER. Inside the RCER, deforestation associated with the OT group is primarily located relatively far from agroextractive villages.

### Temporal Patterns of Deforestation

The analysis of fractional images also allowed the classification of deforestation patches as ´bare soil` or secondary forest. The ´bare soil` class represents very recently cleared land, plantations with less than one year or sites cultivated uninterruptedly for no longer than three years, pastures or villages, whereas the ´secondary forest` class represents abandoned areas or shifting-cultivation fallows that follow the development of manioc plantations. According to interviews with residents, fallow cycles may last, on average, 5.5 years in the study area, but may vary from three to 25 years.

The classification of deforested areas was validated with false color composites of Landsat TM 5 bands 3, 4, and 5 and with extensive field work. Ground-truthing activities were conducted during eight visits to the reserve. These visits included a total of 68 days between May 2007 and May 2008. Land use data were collected in meetings with the residents of agroextractive villages, and representatives of Chico Mendes Institute for Biodiversity Conservation (ICMBio). No specific permits were required for the field studies because all visits to the reserve were conducted in the company of ICMBio staff. Visits to plantations and secondary forests were also accompanied by at least one resident who provided data on the history of land use, the time since occupation, the crops cultivated, the average annual size of the plantations, the fallow cycles, the estimated age of the secondary forest patches, and the fire management of cleared lands. Satellite images were also shown to the residents. This way, it was possible to locate deforested areas that were not visited. Visits to NT patches were conducted during a demographic census by the ICMBio staff. The field studies did not involve endangered or protected species. The data from interviews were double-checked through comparisons with satellite images and with low- altitude flights. Every deforestation patch for which the land cover classification was initially doubtful was visited on the ground.

### Spatial Patterns of Deforestation

Deforestation patches were classified according to elevation [33] and according to their linear distances to: water bodies (rivers and streams), main roads, trails or secondary access roads (*ramais*), municipalities heads, and agroextractive villages, in all analyzed data. Four metrics calculated with Fragstats [34] (number, area, nearest neighbor distance and proximity index) were also used to quantify the spatial patterns of deforestation by the groups. The variables were analyzed with a multivariate discriminant function. This method serves to evaluate the null hypothesis that there are no real groups in the data (i. e., the explanatory variables show similar behavior between the a priori groups) [35]. This analysis assumes that the explanatory variables are normally distributed. Variables that did not follow a normal distribution were log transformed. The variables that still did not follow a normal distribution after transformation were omitted from the discriminant function analysis, but included in the description of the visual patterns. To avoid input data redundancy, a tolerance value of 0.5 was used as the lower threshold for variable acceptance. A significance value of p≤0.001 was used for the analysis.

## Results

### Deforestation Rates

**Figure 2:**
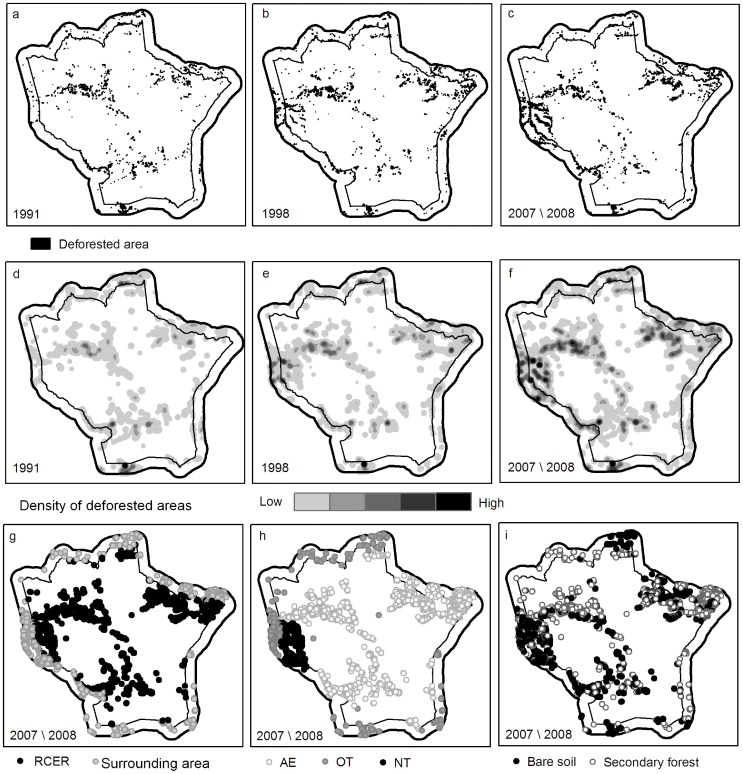
Deforestation in the RCER and its surrounding area in 1991(a), 1998 (b) and 2007/2008 (c). Density of deforested areas in 1991 (d), 1998 (e) and 2007/2008 (f). Deforested areas located in the RCER or in the RCER surrounding area (g). Deforestation patches by causing agents (h). Location of bare soil and secondary forest patches (i).

The analyses of deforestation maps, in 1991, 1998 and 2007/2008 showed that by 2008 human activities had replaced 9,179.2 ha of the original vegetation cover in the RCER and 4,938.5 ha in the area surrounding the reserve. These values represent 1.82% of the total area of the RCER and 2.81% of the surrounding area (Figure 2).

A clearer understanding of the dynamics of forest replacement in the study area was possible through the detailed mapping and classification of deforestation patches as ´bare soil` or ‘secondary forest’. ´Bare soil` represents very recently cleared land, plantations established for less than one year or sites cultivated uninterruptedly for no longer than three years, pastures or villages. In contrast, the ‘secondary forest` represents abandoned areas or shifting cultivation fallows following manioc plantations.

**Figure 3:**
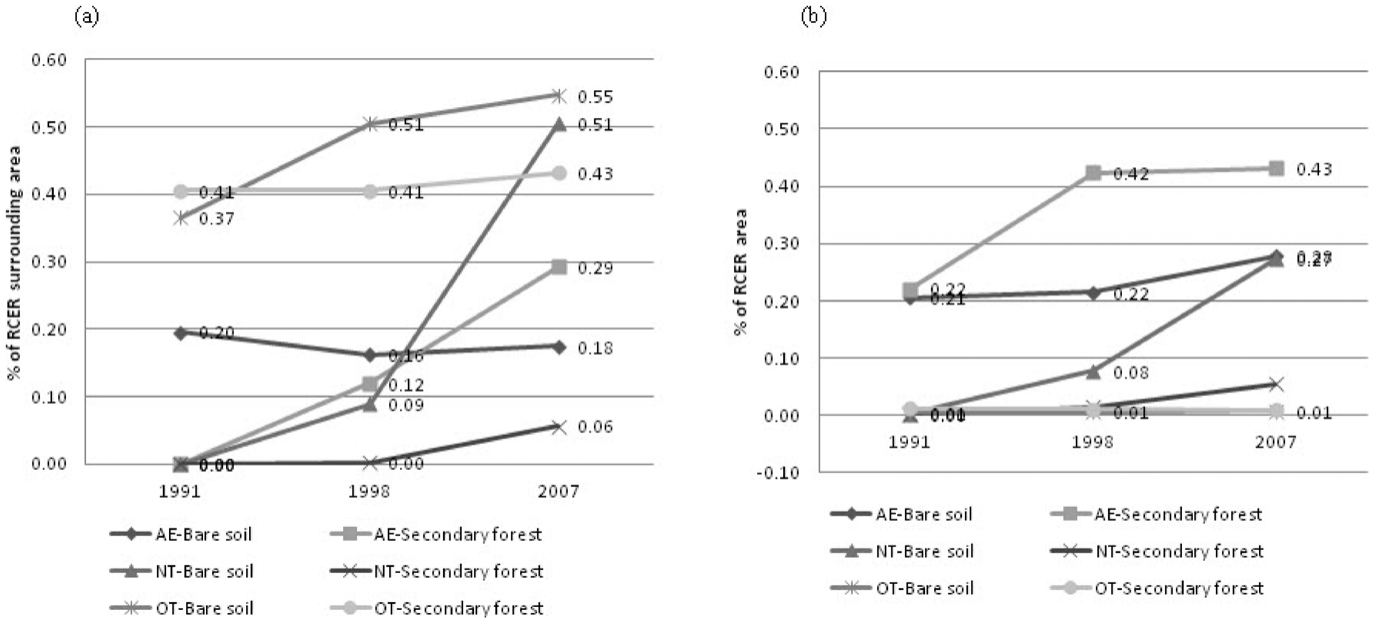
Contributions of the three main agents causing deforestation (non-traditional community (NT), agro-extractive (AE) and others (OT)) to the total area of bare soil and secondary forest in the RCER and in its surrounding area in 1991, 1998 and 2007/2008.

The rate of bare soil formation was higher than that of secondary forest formation in the area surrounding RCER, during 1991–2008. Between 1991 and 1998, the rate of bare soil formation was 0.028% of the area surrounding the RCER per year, whereas the corresponding rate of secondary forest formation was of 0.017% yr^−1^. During 1998–2008, bare soil in the RCER surrounding area increased at a rate of 0.052% yr^−1^, whereas secondary forests increased at a rate of 0.028% yr^−1^ (Figure 3a). Within the RCER, the rate of formation of secondary forests (0.031% yr^−1^) exceeded the rate of formation of bare soil (0.012% yr^−1^) between 1991 and 1998. Between 1998 and 2008, bare soil increased at a higher rate (0.029% yr^−1^) than secondary forests (0.005% yr^−1^).

A better understanding of deforestation was also made possible by the analysis of the contribution of the three primary agents causing deforestation (AE, OT and NT) to the percentages of use and to the rates of bare soil and secondary forest formation in the study area (Figures 3a, b). OT was the most representative group in the area surrounding the RCER in 1991 and 1998. AE showed the highest proportions of bare soil and secondary forest in the RCER in 1991, 1998 and 2007 (Figures 3a, b). In 2007, the proportion of bare soil associated with NT (0.50%), in the area surrounding the RCER, exceeded the proportion of bare soil associated with all other groups except OT (0.54%). The increasing rate of deforestation in the area surrounding the RCER between 1991 and 2008 was primarily associated with the expressive rate of bare soil formation by the NT group between 1998 and 2008 (0.034% of the area surrounding the RCER per year). The increasing deforestation rates within the RCER were also primarily associated with higher rates of bare soil formation by NT during the 1998–2007 period (0.022% yr^−1^). To a lesser extent, the increasing deforestation rates were also associated with the formation of bare soil by the AE group at a rate of 0.007% of the RCER area per year. A decreasing rate of secondary forest formation by the AE group in the RCER area (from 0.029% yr^−1^ to 0.001% yr^−1^) during the second period of the study is also of interest.

### Spatial Patterns of Deforestation

Deforestation patches associated with AE were more numerous on the data analyzed than those associated with the other two groups (Table 1). Inside the RCER, the average size of deforestation patches associated with AE was smaller in 1991 and 1998 than the average size of clearings by the other two groups. In 2007/2008, the average size of clearings by the OT group was smaller than the average size of clearings by the AE group due to the abandonment and reduction of the area of mining sites or to increased cloud cover over the Amazon River islands and in the northern part of the reserve, where most deforestation by the OT is located. According to the data analysis, the number of clearings by the NT group in the area surrounding the RCER was smaller than the number of clearings by the two other groups. Inside the RCER, the number of deforestation patches associated with NT was smaller than the numbers associated with AE and OT in 1991. In 1998 and 2007/2008, OT was the least representative group inside the reserve. The patches of deforestation inside the RCER associated with NT showed the greatest average sizes and relatively high values of the proximity index. The high values of the proximity index imply that areas cleared by the NT group tend to aggregate to form larger clusters. In the area surrounding the RCER, the NT group was associated with the greatest average size of deforestation patches in 1998 and 2008 (Table 1).

**Table 1:** Number of deforestation patches (N), their average size (Size), proximity index (Prox), nearest neighbor distance (ENN).

AE	Location	N	Size (ha)	Prox	ENN (m)
**1991**	**RCER**	826	2.5 (±5.2)	6.5 (±21.4)	348.3 (±659.6)
	**SA**	149	2.1 (±3.2)	3.5 (±4.7)	493.6 (±1715.3)
**1998**	**RCER**	955	3.3 (±6.9)	9.2 (±21.8)	251.1 (±526.3)
	**SA**	161	2.8 (±4.8)	6.8 (±19.7)	245.7 (±312.6)
**2008**	**RCER**	836	4.2 (±8.3)	9.2 (±21)	270.5 (±412.4)
	**SA**	239	3.2 (±5.5)	6.4 (±11.7)	231.3 (±403.1)
**NT**		**N**	**Size (ha)**	**Prox**	**ENN (m)**
**1991**	**RCER**	6	3.7 (±5.8)	0.29 (±0.31)	731.1 (±626.5)
	**SA**	1	0.12	0	0
**1998**	**RCER**	70	7.5 (±17.8)	11.1 (±25.3)	252.8 (±295.8)
	**SA**	15	6.8 (±18.5)	6.2 (±6.5)	242.5 (±235.9)
**2008**	**RCER**	97	18.4 (±46.9)	62.1 (±133)	222.2 (±224.2)
	**SA**	54	15.6 (±39.8)	17.7 (±35.9)	223.5 (±263.1)
**OT**		**N**	**Size (ha)**	**Prox**	**ENN (m)**
**1991**	**RCER**	39	3.4 (±5.6)	4.2 (±11.7)	1369 (±2120.3)
	**SA**	204	6.3 (±25.3)	22.9 (±138.1)	443.9 (±764.9)
**1998**	**RCER**	29	3.8 (±7.8)	0.4 (±0.6)	1884.6 (±2181.9)
	**SA**	272	5.7 (±22.4)	9.4 (±24.4)	373.7 (±734)
**2008**	**RCER**	27	3.2 (±6.7)	0.38 (±0.8)	2929.9(±5957)
	**SA**	192	8.7(±27.4)	16.1 (±36)	468 (±1168.4)

**Figure 4:**
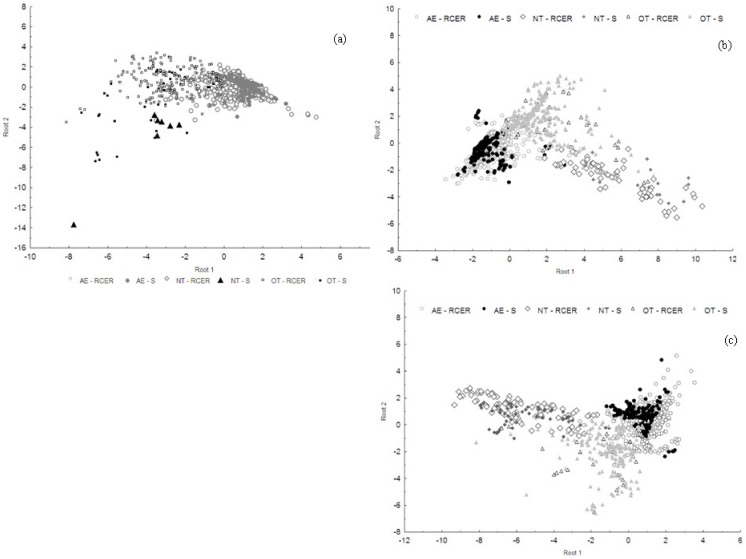
Discriminant function analyses differentiating deforestation spatial patterns by AE, NT and OT inside the RCER and in the RCER surrounding area (S) in 1991 (a), 1998(b) and 2007/2008 (c).

A further analysis of the patterns of deforestation by the three groups showed that the AE group is the least heterogeneous group. A single group associated with deforestation caused by AE inside the RCER and in its surrounding area was identified in 1991, 1998 and in 2007/2008. The same pattern is observed for deforestation caused by NT inside the RCER and in its surrounding area. In contrast, the OT group is the most heterogeneous and least representative group. Descriptive patterns of this group overlap with those of AE and NT (Figures 4a, b, c). The OT group could potentially be divided into other subgroups, and these subgroups would require further investigation. For 1991, the two variables (Axis 1 and 2) in the multivariate discriminant function analysis that best explain the distinctiveness of the deforestation patches associated with the three groups are the distances to any fluvial access and the distance between deforested patches and other vegetation types than dense ombrophilous forest (upland forest) (e. g. flooded forests, inundated savannas and upland forests and open ombrophilous forest). For 1998, the principal variables explaining the distinctiveness of the groups are the distances between deforested patches and other vegetation types and distance to any access (fluvial or terrestrial). For 2007/2008, the main variables explaining the distinctiveness of the groups are the distances to water access, the distances between deforested patches and other vegetation types than upland forests (Table 2).

**Table 2:** Correlation matrix of variables used in the multivariate discriminant function analysis to differentiate deforestation caused by AE, NT and OT in 1991, 1998 and 2007/2008.

	1991		1998		2007/2008	
Variable	Axis 1	Axis 2	Axis 1	Axis 2	Axis 1	Axis 2
**Area**					**−0.109658**	**0.008756**
**Proximit Indexy**	**−0.034651**	**0.243246**	**−0.015238**	**−0.056613**	**−0.148585**	**0.165721**
**Neighboord**	**−0.138946**	**−0.453934**	**0.079136**	**0.071881**	**0.012402**	**−0.139182**
**distance to road**	**0.020321**	**0.066654**	**−0.098044**	**0.149326**	**0.073256**	**0.06726**
**distance to secondary roads**	**0.086162**	**0.187847**	**−0.413649**	**0.238567**	**0.204088**	**−0.196119**
**distance to main rivers**	**−0.100146**	**0.293467**	**−0.000106**	**0.217821**	**0.056753**	**−0.186297**
**distance to any fluvial access**	**−0.533715**	**0.316146**	**0.232069**	**0.468568**	**−0.085927**	**−0.667105**
**distance to any access (terrestrial or fluvial)**	**−0.503684**	**0.249871**	**0.164824**	**0.550004**	**−0.042391**	**−0.649891**
**distance to extractivists` villages.**	**−0.430331**	**0.224412**	**0.357369**	**0.384927**	**−0.309962**	**−0.447015**
**distance tovegetation types**	**−0.41678**	**−0.642048**	**0.692978**	**−0.477713**	**−0.852393**	**0.248494**
**distance to municipality heads**	**−0.045537**	**0.211869**	**−0.189318**	**0.279947**	**0.338565**	**−0.156118**
**elevation**	**−0.482771**	**0.025274**	**0.302827**	**0.246627**	**−0.220432**	**−0.483216**
Eigen-	**2.242621**	**0.284196**	**3.251465**	**1.056399**	**4.060145**	**0.801868**
Chi-Sqr.	**1953.556**	**527.781**	**3589.97**	**1444.401**	**3525.974**	**1234.943**
df	**44**	**30**	**55**	**40**	**60**	**44**
**p-level**	**0**	**0**	**0**	**0**	**0.000000**	**0.000000**

Clearings for agricultural purposes, by all groups (AE, NT and OT), are located exclusively in dense ombrophilous forest due to the fertile soils associated with this vegetation type and shadow conditions. The distance from deforested patches to other vegetation types than the dense ombrophilous forest is an important variable to explain the distinctiveness among the groups because the AE group usually establishes settlements on the border between two or more vegetation types and at relatively lower elevations. For this reason, deforestation patches by AE is located in closer proximity to other vegetation types than NT and OT.

Deforestation associated with the AE is clearly located close to agroextractive villages and far from municipality centers, whereas the opposite pattern is observed for deforestation by the NT group. Patches of deforestation associated with NT are located at higher elevations (from 35 to 171 m) and in close proximity to roads. Clearings by NT are located in upland forests (dense ombrophilous forest) distant from the borders of other vegetation types. The deforestation polygons associated with this group are located far from agroextractive villages and in close proximity to secondary roads and to the municipal center of *Laranjal do Jarí* (Table 2).

OT is a more heterogeneous group. This group includes abandoned mining sites north of the reserve, and clearings associated with agriculture and with cattle and timber production on islands in the Amazon river. They are also located near the village of *Jarilândia*, south of the reserve in the municipality of *Vitória do Jari*, and they may be associated with higher-income farmers settled near road BR-156. The deforestation patches associated with OT are located primarily in the area surrounding the RCER, but may also be found in upland forests. They may also be located on islands in the Amazon River, at lower elevations, and in the northern part of the reserve.

## Discussion

The total deforested area calculated in this study differs significantly from the estimates developed by the PRODES Project (*Projeto de Monitoramento do Desmatamento na Amazônia Legal*) [16]. The deforestation value calculated in this study was 3.72 times higher than the official estimates for the RCER over the 17 year time frame of that analysis. In the area surrounding the RCER, the deforestation value calculated in this study was 3.57 times higher than the total deforested area estimated by PRODES for the same period. The PRODES monitoring program has been useful for detecting deforestation on agricultural frontiers, such as those in Mato Grosso state and in eastern Pará state. These results need to be explored further in other areas if protected areas in the Brazilian Amazon are to be included in national programs for reducing emissions from deforestation and forest degradation [11] due to their implications for CO_2_ baseline estimates. Refinements to the PRODES methodology, such as the one adopted in this work, are necessary to detect land use change in more conservative scenarios. These scenarios include situations in which human population densities are small. They also include situations in which deforestation patches are small, scattered or associated with vegetation types such as upland savannas and inundated savannas rather than with forests.

Clearings detected by the PRODES methodology are restricted to ombrophilous forests. A low resolution polygon mask used by this Program, with scales varying from 1∶500,000 to 1∶1,000,000, filters out deforestation in other vegetation types and in areas of contact between two or more vegetation types. In the RCER, deforestation by extractivists occurs primarily on the border of two or more vegetation types or is associated with patches of forest surrounded by other vegetation types and can be smaller than 6.25 ha. Refinements to the PRODES methodology allowed the identification of deforestation patches as small as 0.09 ha, in all vegetation types in the RCER. A classification of forest clearings associated with distinct social groups in the RCER and its surrounding area was only possible as a result of refinements to the PRODES methodology.

The deforestation numbers in the study area (1.82% of the total RCER area and 2.81% of the area surrounding RCER) are similar to the average deforestation associated with protected areas in the Brazilian Amazon [8]. A closer analysis of the contribution of the three principal social groups occupying the study area AE, OT and NT to the formation of bare soil and secondary forest provided additional evidence about the spatial and temporal patterns of deforestation in the protected area and in the area surrounding the RCER. Increasing rates of deforestation in the RCER and its surrounding area are primarily associated with increasing rates of bare soil formation by NT. This group tends to use their cultivated areas uninterruptedly, with no fallows, and the cultivated sites are usually abandoned after the soil becomes impoverished. The patches of deforestation associated with NT show the greatest average sizes. They also show relatively high values of the proximity index and tend to aggregate to form larger clusters. Deforestation associated with NT is located near to access roads, which facilitate the marketing of their production. Approximately 62% of the NT occupants in the RCER live in urban areas [36] and land use and occupation of the RCER by NT is illegal. Deforestation rates in the RCER could be greatly reduced with an effective regulatory enforcement by the public authorities over the settlement of NT inside this protected area.

The spatial patterns of deforestation by agroextractivists clearly differ from those associated with the NT and OT groups. Despite the higher observed proportion of secondary forests associated with AE, the rate of bare soil formation by this group has increased within the RCER. A reduction in the rate of secondary forest formation by this group within the RCER was also observed. One cause of the increasing rates of deforestation and decreasing rates of secondary forest formation by agroextractivists is the lack of technical assistance for agricultural production. According to the RCER utilization plan, agroextractivists are allowed to increase their crop sizes at the rate of one additional ha yr^−1^. In interviews, members of the AE group have reported that they abandon cultivation sites after the third cycle of manioc cultivation and return to the same site only after the completion of a five year to six- year fallow cycle. The shifting mosaic of crops and fallow areas associated with the AE group results primarily from slash-and-burn techniques. These techniques impoverish the soil after short periods of land use. The same site is not cultivated by an AE family for more than three years. Improved agricultural techniques could ensure that the same site was cultivated for a longer period; thus, preventing deforestation of new areas. Increasing deforestation rates by the AE group have also resulted from migrations following the provision of public services (power, transportation, health services and education) by the municipal and state governments inside the RCER and from increasing population densities of extractivists in the RCER area [27], and from the low value added that is associated with the Brazil nut production chain. AE residents have employed forest management practices with Brazil nut extraction, which favors the perpetuation of forest stands in the long-term. High densities of Brazil nut trees, as high as 104 individuals per hectare, were observed in cultivation sites by agroextractivists [37]. This fact may be associated with dispersion of seeds by agoutis who are attracted by the mature crop [38], [39], and to the abandonment of sites and protection of secondary forests that are naturally enriched with Brazil nut trees by extractivists who recognize the potential extractive value of these sites.

No significant differences were observed in the spatial patterns of deforestation caused by AE, NT or OT inside or outside the RCER. This result implies that the spatial patterns of deforestation by the three groups are not determined by the protected areás boundaries, but by the land management practices adopted by each group. Although the spatial patterns of deforestation seems to be less determined by the protected areás existing boundaries, the protected area creation has provided greater tenure security for the local extractive communities [20] in a area of low development pressure [8]. Historically, the local extractive communities have employed agricultural management practices that are less detrimental to biodiversity. They have also actively contributed to the RCER implementation. AE patches of deforestation are located near villages and far from areas cultivated by nontraditional groups. This difference reflects the tendency of the members of the AE group to protect their ´*colocações* `. It also prevents the deforestation of the areas they occupy, by the non-traditional group of illegal occupants.

Invasion of protected areas and fine-grained spatial patterns of deforestation are rarely quantified in the Brazilian Amazon [14], but their effects are cumulative and should not be overlooked since they may be an obstacle to the implementation of protected areas in the long-term. Threats to protected areas are conspicuous and may be overlooked or poorly identified in other protected areas of low development pressure due to the lack of detailed maps and analysis of the spatial patterns of deforestation.

## References

[1] Parker C, Mitchell A, Trivedi M, Mardas N (2008) The Little REDD Book. Oxford, UK: The Global Canopy Programme.

[2] Kintisch E (2009) Deforestation moves to the fore in Copenhagen. Science 326: 1465. 20007869 10.1126/science.326.5959.1465

[3] Nepstad D, Soares-Filho BS, Merry F, Lima A, Moutinho P, et al (2009) Environment. The end of deforestation in the Brazilian Amazon. Science 326: 1350–1351. 19965742 10.1126/science.1182108

[4] Putz FE, Zuidema PA, Pinard MA, Boot RGA, Sayer JA, et al (2008) Improved tropical forest management for carbon retention. PLoS Biol 6: 166. 10.1371/journal.pbio.0060166 PMC2459208 18630991

[5] Walker R, Moore NJ, Arima E, Perz S, Simmons C, et al (2009) Protecting the Amazon with protected areas. Proc Natl Acad Sci 106: 10582–10586.11. 19549819 10.1073/pnas.0806059106 PMC2705550

[6] Soares-Filho B, Moutinho P, Nepstad D, Anderson A, Rodrigues H, et al (2010) Role of Brazilian Amazon protected areas in climate change mitigation. Proc Natl Acad Sci 107: 10821–10826.12. 20505122 10.1073/pnas.0913048107 PMC2890753

[7] Galford GL, Melillo JM, Kicklighter DW, Cronina TW, Cerri C, et al (2010) Greenhouse gas emissions from alternative futures of deforestation and agricultural management in the southern Amazon. Proc Natl Acad Sci 107: 19649–19654.13. 20651250 10.1073/pnas.1000780107 PMC2993363

[8] Bruner AG, Gullison RE, Rice RE, da Fonseca GAB (2001) Effectiveness of parks in protecting tropical biodiversity. Science 291: 125–128. 11141563 10.1126/science.291.5501.125

[9] Nepstad D, Schwartzman S, Bamberger B, Santilli M, Ray D, et al (2006) Inhibition of Amazon deforestation and fire by parks and indigenous lands. Conserv Biol 20: 65–73. 16909660 10.1111/j.1523-1739.2006.00351.x

[10] Joppa LN, Bane SR, Pimm SL (2008) On the protection of protected areas. Proc Natl Acad Sci U S A 105: 6673–6678. 18451028 10.1073/pnas.0802471105 PMC2365567

[11] Barber CP, Cochrane MA, Souza Jr C, Verissimo A (2012) Dynamic performance assessment of protected areas. Biol. Conserv 149: 6–14.

[12] Andam S, Ferraro P, Pfaff A, Sanchez-Azofeifa G, Robalino J (2008) Measuring the effectiveness of protected area networks in reducing deforestation. Proc Natl Acad Sci USA 105: 16089–1609.10. 18854414 10.1073/pnas.0800437105 PMC2567237

[13] Fearnside PM (2003) Conservation Policy in the Brazilian Amazonia: understanding the dilemmas. World Dev 32: 757–77914.

[14] Ricketts TH, Soares-Filho B, Fonseca GAB, Nepstad D, Pfaff A, et al (2010) Indigenous Lands, Protected Areas, and Slowing Climate Change. PLoS Biol 8: 3. 10.1371/journal.pbio.1000331 PMC2838743 20305712

[15] Instituto Brasileiro dos Recursos Naturais Renováveis Reserva Extrativista do Rio Cajarí- Plano de utilização. Available: http://www.ibama.gov.br/resex/cajari/plano.htm. Accessed 2011 Aug 1.

[16] Instituto Nacional de Pesquisas Espaciais (2012) PRODES: Assessment of deforestation in Brazilian Amazonia. Available: http://www.obt.inpe.br/prodes/. Accessed 2012 Jun 6.

[17] Borges SH, Iwanaga S, Moreira M, Durigan CC (2007) Uma análise geopolítica do atual sistema de unidades de conservação na Amazônia Brasileira. Política Ambiental 4.

[18] Peres CA, Barlow J, Laurance WF (2006) Detecting anthropogenic disturbance in tropical forest. Trends Ecol Evol 21: 227–229. 16697907 10.1016/j.tree.2006.03.007

[19] Secretaria de do Meio Ambiente do Estado do Amapá (2011) Relatório Técnico do Desmatamento no Estado do Amapá referente aos anos de 2009 a 2010 Macapá: SEMA.

[20] Ioun C, Aoki Y (2007) Brazilian experiences in sustainable reserves. Brasília: Conservação Internacional 94 p.

[21] Brasil (1997) Decreto s/n de 30 de setembro de 1997. Diário Oficial da União n° 189.

[22] Instituto de Pesquisas Científicas e Tecnológicas do Estado do Amapá (1997) Zoneamento Ecológico e Econômico (ZEE). Escala 1: 1.000.000. Amapá: IEPA.

[23] Souza EB, Cunha AC (2010) Climatologia de precipitação no Amapá e mecanismos climáticos de grande escala in Tempo, Clima e Recursos Hídricos. Technical report. Macapá: IEPA.

[24] Picanço JRA (2005) Reserva Extrativista do Rio Cajari: verso e reverso da territorialização no sul do Amapá/José Reinaldo Alves Picanço. Master thesis. Univesidade Federal do Rio Grande do Norte. 158 p.

[25] Brasil (2008) Atlas de Unidades de Conservação do Estado do Amapá. Macapá: SEMA.

[26] Brasil (1990) Decreto n° 99.145, de 12 de março de 1990. Cria a Reserva Extrativista do rio Cajari. Available: http://www.ibama.gov.br/siucweb/mostraDocLegal.php?seq_uc=673eseq_tp_documento=3eseq_finaliddoc=7. Accessed 2006 Nov 13.

[27] Picanço JRA (2009) Desenvolvimento, sustentabilidade e conservação da biodiversidade na Amazonia: a produção familiar agroextrativista em áreas protegidas no sul do Amapá. Dissertation. Universidade Federal do Rio Grande do Norte. 381 p.

[28] Sistema Nacional de Unidades de Conservação (2000) SNUC.Lei 9985, 18 de julho de 2000.

[29] Shimabukuru YE, Novo EM, Ponzoni FJ (1998) Índice de Vegetação e Modelo Linear de Mistura Espectral no Monitoramento da Região do Pantanal. Brasília: Pesquisa Agropecuária Brasileira33: 1729–1739.

[30] Shimabukuru YE, Duarte V, Dos SantosJR, Mello EMK, Moreira JC (1999) Levantamento de áreas desflorestadas na Amazônia através de processamento digital de imagens orbitais. Rio de Janeiro: Floresta e Ambiente. 6: 38–44.

[31] Instituto Nacional de Pesquisas Espaciais (2008) Classificação de Imagens. Available: http://www.dpi.inpe.br/spring/usuario/c_clapix.htm. Accessed 2009 Jan 10.

[32] Lins C (2001) Jarí: 70 anos de história. Rio de Janeiro: Dataforma.

[33] Nasa (2005) National Aeronautics and Space Administration. Shuttle Radar Topography Mission (SRTM) Available: http://www2.jpl.nasa.gov/srtm. Accessed 2005 Feb 11.

[34] McGarigal K, Cushman SA, Neel MC, Ene E (2002) FRAGSTATS: Spatial Pattern Analysis Program for Categorical Maps. Computer software program produced by the authors at the University of Massachusetts, Amherst. Available: http://www.umass.edu/landeco/research/fragstats/fragstats.html.

[35] Manly BJF (2008) Métodos estatísticos multivariados: uma introdução. Porto Alegre: Bookmam. 229 p.

[36] Instituto Chico Mendes de Conservação da Biodiversidade (2007) Relatório Técnico da Força Tarefa realizada na parte oeste da Reserva Extrativista do Rio Cajarí. Macapá: ICMBio.

[37] Paiva PM, Guedes MC, Funi C (2010) Brazil nut conservation through shifting cultivation. For Ecol Manage 261: 508–514.

[38] Cotta JN, Kainer KA, Wadt LO, Staudhammer CL (2008) Shifting cultivation effects on Brazil nut (*Bertholletia excelsa*) regeneration. For Ecol Manage 256: 28–35.

[39] Myers GP, Newton AC, Melgarejo O (2000) The influence of canopy gap size on natural regeneration of Brazil nut (*Bertholletia excelsa*) in Bolivia. For Ecol Manage 127: 119–128.

